# HIV Infection Rates and Risk Behavior among Young Men undergoing community-based Testing in San Diego

**DOI:** 10.1038/srep25927

**Published:** 2016-05-16

**Authors:** Martin Hoenigl, Antoine Chaillon, Sheldon R. Morris, Susan J. Little

**Affiliations:** 1 Division of Infectious Diseases, University of California San Diego, San Diego, California, United States; 2 Section of Infectious Diseases and Tropical Medicine, Department of Internal Medicine, Medical University of Graz, Graz, Austria; 3 Division of Pulmonology, Department of Internal Medicine, Medical University of Graz, Graz, Austria

## Abstract

Approximately 80% of new HIV infections in the United States occur in men. Four out of five men diagnosed with HIV infection are men who have sex with men (MSM), with an increasing proportion of young MSM (i.e. ≤24 years of age). We performed a retrospective analysis 11,873 cisgender men participating in a community based HIV screening program in San Diego between 2008 and 2014 to characterize the HIV prevalence and sexual risk behaviors among young men. In young heterosexual men HIV prevalence was lower compared to heterosexual men between 25 and 49 years of age (0.3% *vs.* 1.4%, p = 0.043). Among young MSM, HIV prevalence was 5.5%, per test positivity rate 3.6%, and HIV incidence 3.4 per 100 person years (95% CI 2.2–5.4). Per test positivity rate (p = 0.008) and incidence (p < 0.001) were significantly higher among young MSM than among MSM above 24-years of age. Young MSM diagnosed with HIV infection reported significantly more serodiscordant condomless anal intercourse, bacterial sexually transmitted infections, and higher rates of methamphetamine and gamma hydroxybutyrate use when compared to young MSM who tested negative. In conclusion, young MSM are particularly vulnerable to HIV infection and may represent ideal candidates for targeted prevention interventions that increase testing uptake and/or decrease the risk of acquiring HIV infection.

Men account for about 80% of new HIV infections in the United States^1^. Four out of five men diagnosed with HIV infection are men who have sex with men (MSM). While most HIV infections are still diagnosed in men above 25 years of age, HIV incidence and prevalence among young MSM (i.e. ≤24 years of age) is increasing^1^,^2^. In particular black young MSM are disproportionately affected by HIV, accounting for half of new HIV diagnoses among young MSM^2^,^3^,^4^. While a number of studies have evaluated prevalence and incidence of HIV infection among young MSM in settings where African Americans account for a significant proportion of the population^3^,^4^,^5^,^6^,^7^,^8^,^9^,^10^, fewer studies have characterized HIV risk among young MSM in other settings of the United States^11^. The objective of this study was to examine prevalence and incidence of HIV, as well as racial disparities and risk behavior, all stratified by age, among men undergoing community-based testing in San Diego, where African Americans represent less than 7% of the population.

## Results

HIV prevalence and incidence, per test positivity rates and demographics in MSM and heterosexual men stratified by age are depicted in Table 1. Young MSM were more likely to report Hispanic ethnicity and less likely white race when compared to older MSM (p < 0.001). Among young MSM, HIV prevalence was 5.5% (95% CI 4.4–6.6%), and per test positivity rate was 3.6% (95% CI 2.9–4.3%). HIV prevalence did not differ between young MSM and MSM between 25 and 49 years of age (5.5% *vs.* 4.9%, n.s.), but was significantly higher among young MSM than among MSM 50 years or older (5.5% *vs.* 2.5%, p < 0.001). When compared to MSM above 24 years of age per test positivity rate was significantly higher among young MSM (3.6% *vs.* 2.2%, p < 0.001). HIV incidence was 3.4 per 100 person years (95% CI 2.2–5.4) among young MSM and therefore significantly higher than among MSM above 24 years of age (HIV incidence 1.6, 95% CI 1.3–2.0; p < 0.001). In contrast, HIV prevalence was lower among young heterosexual men when compared to heterosexual men between 25 and 49 years of age (0.3% *vs.*1.4%, p = 0.043), and incidence or per-test positivity did not differ by age.

Risk Behaviour reported for the 12 months prior to diagnosis/last HIV test in MSM and heterosexual men ≤24 years and ≥50 years of age (i.e. two groups with significantly differing HIV prevalence among MSM) are depicted in Table 2. Compared to MSM ≥50 years of age, young MSM reported more condomless anal intercourse (p < 0.001), bacterial sexually transmitted infections (STI) (p < 0.001) and non-injection stimulant drug use (p < 0.001), but total number of partners did not differ. Young heterosexual males reported also more female partners and non-injection stimulant drug use when compared to heterosexual men ≥50 years of age. When compared to MSM between 25 and 49 years of age young MSM reported more condomless receptive anal intercourse (CRAI) (p < 0.001), while rates of condomless insertive anal intercourse, bacterial STI, non-injected stimulant drug use and also numbers of partners did not differ significantly (data not shown, risk behaviour for the overall MSM cohort – not stratified by age - published previously^19^). Markedly lower HIV prevalence and risk behavior was observed in young heterosexual men, resembling those in young cisgender women (HIV prevalence in women 0.4%, median 2 male partners, CRAI in 12.3%, condomless receptive vaginal intercourse in 83%, bacterial STI in 6.6%, non-injection stimulant drug use in 25.4% and IDU in 6%; detailed data not shown). Overall, in MSM and heterosexual men condom use did not differ significantly over the years of the study, and no increase in condomless sex was observed over the study period (detailed data not shown).

For young MSM risk behaviors in those newly diagnosed with HIV infection versus those with negative HIV test results are displayed in Table 3. Median age of those 90 young MSM newly diagnosed with HIV infection was 22 years (IQR 21–23 years). Overall, 40/90 (44%) reported Hispanic ethnicity, 54 (60%) white race, and 3 (3%) black race. Race and ethnicity distributions among young MSM diagnosed with HIV infection did not differ significantly from young MSM with negative HIV test results. Young MSM diagnosed with HIV infection had more condomless anal intercourse and in particular also more CRAI with HIV positive partners (p < 0.001) when compared to those who tested negative (Table 3). In addition bacterial STI (p = 0.002) as well as use of methamphetamine (p < 0.001) and gamma hydroxybutyrate (GHB) (p < 0.001) were significantly more frequent reported by young MSM diagnosed with HIV. San Diego Early test (SDET) risk behavior scores were significantly higher in young MSM diagnosed with HIV compared to those who tested negative (median 3, IQR 1–7 *vs.* median 2, IQR 0–5; p < 0.001).

## Discussion

Our study found that young MSM had the highest incidence of HIV when compared to other MSM, and heterosexual men. While the finding that young MSM are at particular risk for HIV infection confirms results of previous studies^3^,^4^,^5^,^7^,^8^,^10^,^12^,^13^,^14^, it is important to highlight that our findings are not being driven by young African American men as in other parts of the country; only 3% of young MSM diagnosed with HIV reported black race in our cohort (in the most recent census in 2010, 5% of the population of San Diego county was Black or African American). In contrast, Hispanics accounted for 44% of HIV diagnoses among young MSM (Hispanics represented 31.6% of the population of San Diego county). While HIV prevalence rates were about the same in young MSM and those between 25 and 49 years of age (5.5% vs. 4.9%, n.s.), young MSM had significantly fewer testing encounters (25% vs. 35% were repeat testers with the Early Test, p < 0.001), which resulted in higher per-test positivity rates among young MSM (1 out of 28 tests positive) when compared MSM 25 years or older (1 out of 45 tests positive, p < 0.001). Interestingly, those young MSM who underwent voluntary repeat testing were at particular risk for acquiring HIV infection with an incidence rate of 3.4 per 100 person years which was twice as high as the one observed among MSM above 25 years.

When compared to MSM 50 years or older (HIV prevalence 2.5%), significantly higher rates of unprotected anal intercourse, bacterial STI and stimulant substance use were reported among young MSM, whereas total number of partners during the last 12 months did not differ. While higher rates of stimulant substance use may explain the higher rates of condomless anal sex among young MSM^15^, the observation of a recent study that condom use errors and problems, such as breakage and slippage were very common among young MSM^16^, may offer additional explanation. However, individual risk behaviors did not fully explain HIV incidence among young MSM which may make a cause for the need to address sociodemographic and structural-level factors in public health interventions targeted toward young MSM^5^.

In contrast to MSM, young heterosexual men had lower HIV prevalence compared to heterosexual men between 25 and 49 years, and similar prevalence compare to those 50 years or older. Interestingly, the main difference in risk behavior between young heterosexual men and those 50 years or older, besides higher stimulant substance use, was higher numbers of partners, while overall rates of unprotected sex did not differ.

Young MSM diagnosed with HIV infection had more condomless anal intercourse and in particular also more CRAI with serodiscordant partners when compared to young MSM with negative HIV test results. In addition self-reported bacterial STI as well as use of methamphetamine and GHB were significantly more frequent in young MSM diagnosed with HIV. Our findings indicate that at risk young MSM may be ideal candidates for targeted prevention interventions that increase condom use and/or preexposure prophylaxis (PrEP). PrEP with daily tenofovir and emtricitabine has been proven to reduce the acquisition of HIV among MSM by 68% in those who had 90% self-reported adherence^17^, and over 90% in those with high levels of adherence by TDF drug levels^18^. Whether high TDF adherence rates can be achieved among young MSM, in particular those with substance use, remains to be determined in future trials.

Our study has several limitations including its single-center and retrospective design. Additionally, incidence rates observed among young MSM in this study, although high, were still markedly lower than those observed previously among some cohorts of young black MSM^4^,^8^. Together with our previous finding that black MSM, when compared to white MSM, are more likely to be diagnosed at a late stage of HIV infection^19^, these findings may indicate that the low number of young black MSM diagnosed with HIV may be a result of infrequent testing.

In conclusion, our study indicates that young MSM across race are particularly vulnerable to HIV infection and may represent ideal candidates for targeted prevention interventions to encourage use of condoms or PrEP, increase HIV testing and/or decrease risk behaviors.

## Material and Methods

In this cohort study we analyzed HIV prevalence and incidence, per test positivity rate, and race/ethnicity in 11,873 cisgender men who enrolled in the “Early Test”, a voluntary, community-based, confidential acute and early HIV-screening program in San Diego, California. The “Early Test” provides point-of-care (POC) rapid HIV serologic testing followed by reflex HIV nucleic acid amplification testing (NAT) in all antibody (Ab)-negative persons^19^,^20^,^21^,^22^,^23^. Between April 2008 and July 2014, the “Early Test” program provided no cost HIV screening to approximately 4,000 individuals per year at five testing sites in central San Diego plus additional mobile testing sites (including sites targeting MSM at the Lesbian, Gay, Bisexual, Transgender Center and the Gay Men’s Health Clinic; the AntiViral Research Center (AVRC); substance abuse treatment centers; and special community event venues) in San Diego^21^. Pre-test counseling was provided at each Early Test visit throughout the study period and utilized a client-centered harm reduction model offering personalized risk reduction options^21^. Demographics and behavioral risks for the previous 12 months were collected using a short 19-question survey [focusing primarily on sexual risk behavior, substance use, and sexually transmitted infection (STI) diagnoses]. In repeat testers, data reported at the most recent Early Test encounter were used. Survey questions were assessed and the form filled out by the testing staff before each HIV testing encounter, and data was later entered into the data system (always in duplicate to minimize data entry errors). After testing negative, MSM clients were recommended to come back 3 months later for the next testing encounter, while those who tested positive were referred to care^21^.

Study inclusion was limited to cisgender male adolescents and men ≥13 years of age. MSM were defined as men who reported sexual contact with one or more male partners during the previous 12 months and/or self-identified as MSM. Cisgender men not reporting sex with men who did not self-identify as MSM were defined as heterosexual men for study purposes.

Overall, the study included 11,873 men that were stratified by age group (≤24 years, 25 to 49 years, and ≥50 years of age) and sexual behavior (men who did or did not report sex with men). We calculated HIV prevalence and incidence, per test positivity rate as well as demographics for MSM and heterosexual men stratified by age group (≤24 years, 25 to 49 years, and ≥50 years of age). HIV Risk behavior for the overall MSM cohort (i.e. not stratified by age) has been published previously^19^. As a second step we compared risk behavior between young men (≤24 years) and older men (≥50 years), to evaluate whether risk behavior may offer explanation for differing prevalence and per test positivity rates. Finally we compared risk behavior among young MSM who tested positive for HIV with those young MSM who remained negative (factors associated with acute and early HIV infection and risk behaviors for the overall cohort of MSM and also transgender men and women– not stratified by age – have been published previously^19^,^24^).

San Diego Early Test (SDET) scores were calculated as a measure of HIV associated risk behavior^25^, and compared between young MSM newly diagnosed with HIV infection and those with negative test results. The SDET score was recently developed to better estimate incident HIV infection risk in MSM, as assessed by a score ranging from 0 to 10. The score is based on key risk variables that predict risk of HIV acquisition among MSM: condomless receptive anal intercourse (CRAI), number of male partners, and self-reported bacterial STI. While the SDET score captures changes in sexual risk behavior associated with substance use, the score remains independent of specific regional/individual drug use behavior (e.g. methamphetamine use) and thus may be broadly applicable to MSM populations^15^.

The UCSD Human Research Protections Program approved the study protocol, consent and all study related procedures. All study participants provided voluntary, written informed consent before any study procedures were undertaken. The methods were carried out in accordance with the approved guidelines. For statistical analysis SPSS 21 (SPSS Inc., Chicago, IL, USA) was used. Proportions were compared using Fishers exact test or Chi-squared test including Yates correction, as appropriate, and two sided p-values were displayed. Continuous variables were compared using Mann Whitney U test due to non-normality of distribution. 95% confidence intervals (CI) were calculated for all rates, using Poisson confidence intervals specifically for incidence rates.

## Additional Information

**How to cite this article**: Hoenigl, M. *et al.* HIV Infection Rates and Risk Behavior among Young Men undergoing community-based Testing in San Diego. *Sci. Rep.* **6**, 25927; doi: 10.1038/srep25927 (2016).

## Figures and Tables

**Table 1:** HIV Prevalence and Incidence, per Test Positivity and Demographics of Study Participants.

*Risk-factor/Risk behavior and Demographics*	*Men who have sex with men (MSM)$*				*Men who have sex with women (heterosexual)*¥			
	Men ≤ 24 years of age (n = 1639)	Men > 24 years and <50 years of age (n = 6146)	Men ≥ 50 years of age (n = 1141)	P value*	Men ≤ 24 years of age (n = 577)	Men > 24 years and <50 years of age (n = 1907)	Men ≥ 50 years of age (n = 460)	P value*
HIV Prevalence	90 (5.5%)	300 (4.9%)	29 (2.5%)	<0.001	2 (0.3%)	26 (1.4%)	3 (0.7%)	n.s.
Acute HIV	20 (1.2%)	67 (1.1%)	6 (0.5%)	—	0	0	0	—
Early HIV	23 (1.4%)	78 (1.3%)	6 (0.5%)	—	0	2 (0.1%)	0	—
Chronic HIV	47 (2.9%)	155 (2.5%)	17 (1.5%)	—	2 (0.3%)	24 (1.3%)	3 (0.7%)	—
Repeat Testers	409 (25.0%)	2145 (34.9%)	400 (35.1%)	<0.001	40 (6.9%)	223 (11.7%)	44 (9.6%)	0.004
Per Test Positivity Rate (number of tests)	3.6% (2482)	2.4% (12,495)	1.2% (2346)	<0.001	0.3% (643)	1.1% (2373)	0.5% (547)	n.s.
HIV Incidence among repeat Testers per 100 person years (number of incident infections)	3.40 (17)	1.79 (81)	0.85 (8)	<0.001	0.0 (0)	0.26 (1)	0.0 (0)	n.s.
*Demographic Data*								
Age (years; median, IQR)	22 (21–23)	33 (29–40)	55 (52–62)	<0.001	22 (20–23)	34 (29–41)	55 (52–60)	<0.001
Hispanic Ethnicity	627/1596 (39.3%)	1667/5991 (27.8%)	121/1103 (11.0%)	<0.001	240/555 (43.2%)	536/1834 (29.2%)	80/443 (18.1%)	<0.001
Race	—	—	—	<0.001	—	—	—	<0.001
White	808/1490 (54.2%)	3883/5793 (67.0%)	979/1107 (88.4%)	—	280/533 (52.5%)	1044/1803 (57.9%)	308/446 (69.1%)	—
Black	102/1490 (6.8%)	333/5793 (5.7%)	27/1107 (2.4%)	—	53/533 (9.9%)	237/1803 (13.1%)	75/446 (16.8%)	—
Asian	165/1490 (11.1%)	410/5793 (7.1%)	19/1107 (1.7%)	—	47/533 (8.8%)	136/1803 (7.5%)	9/446 (2.0%)	—
Pacific Islander	35/1490 (2.3%)	153/5793 (2.6%)	7/1107 (0.6%)	—	5/533 (0.9%)	21/1803 (1.2%)	1/446 (0.2%)	—
Native American	18/1490 (1.2%)	33/5793 (0.6%)	1/1107 (0.1%)	—	19/533 (3.6%)	53/1803 (2.9%)	4/446 (0.9%)	—
Other	362/1490 (24.3%)	981/5793 (16.9%)	74/1107 (6.7%)	—	129/533 (24.2%)	312/1803 (17.3%)	49/446 (11.0%)	—

Abbreviations: IQR, inter-quartile range; n.s.; not significant.

^$^includes men who have sex with men and women

*calculated using Chi-squared test.

^¥^Includes all cisgender men not self identifying as MSM and not reporting sexual intercourse with a male partner during the recent 12 months.

**Table 2:** Risk Behaviour Reported for the 12 months prior to diagnosis/last HIV test in MSM and heterosexual men ≤24 years and ≥50 years of age undergoing community based HIV testing.

*Risk-factor/Risk behavior*	*Prevalence of risk behaviour among cisgender men undergoing community-based voluntary HIV testing*					
	Men who have sex with men ≤24 years of age (n = 1639)	Men who have sex with men ≥50 years of age (n = 1141)	P value*	Heterosexual Men ≤24 years of age (n = 577)¥	Heterosexual Men ≥50 years of age (n = 460)¥	P value*
Total Number of partners (median, IQR)	6 (3–11)	5 (3–12)	n.s.	3 (1–5)	2 (1–3)	<0.001
Intercourse with Male partner	1639/1639 (100%)	1141/1141 (100%)	n.s.	0/577 (0%)	0/460 (0%)	n.s.
Number of Male partners (median, IQR)	5 (3–10)	5 (3–12)	n.s.	—	—	—
Intercourse with Female partner	220/1639 (13.4%)	110/1141 (9.6%)	0.029	537/577 (93.1%)	381/460 (82.8%)	<0.001
Number of Female partners (median, IQR)	2 (1–3)	1 (1–2)	n.s.	3 (1–5)	2 (1–4)	<0.001
Intercourse with Transgender partner	15/1634 (0.9%)	14/1141 (1.2%)	n.s.	13/574 (2.3%)	15/460 (3.3%)	n.s.
Number of Transgender partners (median, IQR)	1 (1–2)	1 (1–1)	n.s.	1 (1–1)	1 (1–1)	n.s.
Intercourse with MSM	100%	100%	n.s.	0%	0%	n.s.
10 or more total partners	522/1634 (31.9%)	398/1139 (34.9%)	n.s.	59/568 (10.4%)	30/449 (6.7%)	0.049
CIAI total	974/1629 (59.8%)	599/1132 (52.9%)	<0.001	96/509 (18.9%)	56/367 (15.3%)	n.s.
CIAI with Female/Transgender	23/215 (10.7%)	18/109 (16.5%)	n.s.	96/509 (18.9%)	56/367 (15.3%)	n.s.
CIAI with Male	969/1629 (59.5%)	588/1132 (51.9%)	<0.001	0	0	n.s.
CRAI	967/1629 (59.4%)	430/1134 (37.9%)	<0.001	1/577 (0.2%)#	0	n.s.
CRAI and 5 or more partners	624/1629 (38.3%)	276/1134 (24.3%)	<0.001	1/577 (0.2%)#	0	n.s.
CPVI	89/216 (41.2%)	78/105 (74.3%)	<0.001	395/518 (76.3%)	288/370 (77.8%)	n.s.
CRAI with HIV positive	89/1515 (5.9%)	42/1104 (3.8%)	0.021	0	0	n.s.
Bacterial STI§	254/1639 (15.5%)	59/1141 (5.2%)	<0.001	19/577 (3.3%)	6/460 (1.3%)	n.s.
Non injection stimulant Drug use (i.e. methamphetamine, cocaine, poppers, GHB, ketamine, XTC)	454/1639 (27.7%)	208/1141 (18.2%)	<0.001	161/577 (27.9%)	66/460 (14.3%)	<0.001
Methamphetamine	97/1639 (5.9%)	53/1141 (4.6%)	n.s.	74/577 (12.8%)	43/460 (9.3%)	n.s.
GHB	67/1639 (4.1%)	25/1141 (2.2%)	0.008	4/577 (0.7%)	1/460 (0.2%)	n.s.
XTC	236/1639 (14.4%)	24/1141 (2.1%)	<0.001	74/577 (12.8%)	4/460 (0.9%)	<0.001
Nitrites, inhaled	216/1639 (13.2%)	146/1141 (12.8%)	n.s.	4/577 (0.7%)	2/460 (0.2%)	n.s.
Cocaine	192/1639 (11.7%)	24/1141 (2.1%)	<0.001	97/577 (16.8%)	29/460 (6.3%)	<0.001
IDU	16/1639 (1.0%)	13/1141 (1.1%)	n.s.	28/577 (4.9%)	21/460 (4.6%)	n.s.

Risk behaviour not stratified by age for the overall MSM cohort including MSM 25–49 years of age has been published previously^19^.

Abbreviations: CIAI, condomless insertive anal intercourse; CPVI, condomless penetrative vaginal intercourse; CRAI, condomless receptive anal intercourse; GHB, gamma hydroxybutyrate; IDU, injection drug use; IQR, inter-quartile range; MSM, men who have sex with men; n.s.; not significant; STI, sexual transmitted infection; XTC = ecstasy.

*Calculated using Chi-square or Mann Whitney U test.

^#^With Transgender partner.

^§^Syphilis, chlamydia and gonorrhea.

^¥^Includes all males not selfidentifying as MSM and not reporting sexual intercourse with a male partner during the recent 12 months.

**Table 3:** Risk Behaviour in young MSM (≤24 years of age) newly diagnosed with HIV Infection as compared to those with negative HIV Test Results.

*Risk-factor/Risk behavio*	*Prevalence of risk behaviour among cisgender men undergoing community-based voluntary HIV testing*		
	Young MSM with HIV Infection (n = 90)	Young MSM with negative HIV Test Results (n = 1549)	P value*
**Number of Male partners (median, IQR)**	6 (3–16)	5 (3–10)	0.055
**Intercourse with Female partner**	10/90 (11.1%)	210/1549 (13.6%)	n.s.
**10 or more male partners**	36/90 (40.0%)	463/1549 (29.9%)	0.056
**CIAI with Male**	66/88 (75.0%)	903/1541 (58.6%)	0.003
**CRAI**	74/89 (83.1%)	893/1540 (58.0%)	<0.001
**CRAI and 5 or more partners**	45/89 (50.6%)	579/1540 (37.6%)	0.020
**CRAI with HIV positive**	21/79 (26.6%)	68/1436 (4.7%)	<0.001
**Bacterial STI§**	25/90 (27.8%)	229/1549 (14.8%)	0.002
**Non injection stimulant Drug use (i.e. methamphetamine, cocaine, poppers, GHB, ketamine, XTC)**	30/90 (33.3%)	424/1549 (27.4%)	n.s.
**Methamphetamine**	16/90 (17.8%)	81/1549 (5.2%)	<0.001
**GHB**	12/90 (13.3%)	55/1549 (3.6%)	<0.001
**XTC**	15/90 (16.7%)	221/1549 (14.3%)	n.s.
**Nitrites, inhaled**	13/90 (14.4%)	203/1549 (13.1%)	n.s.
**Cocaine**	15/90 (16.7%)	177/1549 (11.4%)	n.s.
**IDU**	2/90 (2.2%)	14/1549 (0.9%)	n.s.
**San Diego Early Test (SDET) Scores (median, IQR)**	3 (1–7)	2 (0–5)	<0.001

Abbreviations: CIAI, condomless insertive anal intercourse; CRAI, condomless receptive anal intercourse; GHB, gamma hydroxybutyrate; IDU, injection drug use; IQR, inter-quartile range; MSM, men who have sex with men; n.s., not significant; STI, sexual transmitted infection

*Calculated using Chi-square or Mann Whitney U test. P-value displayed if p<0.06.

^§^Syphilis, chlamydia and gonorrhea.

XTC, ecstasy.
